# A Treatise on the Medicinal Leech, Including Its Medical and Natural History, with a Description of Its Anatomical Structure: Also, Remarks upon the Diseases, Preservation, and Management of Leeches

**Published:** 1816-12

**Authors:** 


*A Treatise on the Medicinal Leech, including its Medical and Natural History, with a Description of its Anatomical Structure: also, Remarks upon the Diseases, Preservation, and Management of Leeches.* By JAMES RAWLINS JOHNSON, M. D. F. L. S. &c. Illustrated with two Engravings. Octavo, pp. 147, London, 1816.

AMONG the few individuals of the animal kingdom to which man has recourse, in remedying the various injuries and diseases entailed upon him, sometimes by the constitution of his organs, but more frequently by the defects of his physical education, or the noxious influence of his habits and pursuits, none occupies a more important rank than the Medicinal Leech; none, perhaps, with the exception of the *lytta*, is capable of application so beneficial and extensive. The present scarcity of the leech, in this island, which may be traced partly to the destruction of its accustomed haunts by the cultivation of our waste lands, and partly to the increased employment of the animal in the treatment of diseases, unfortunately, renders us dependent on a foreign and precarious source of supply;* and many persons are excluded, by its consequent high price, from the benefits of the operation, or purchase them by sacrifices and privations which, in the time of sickness, they are but ill prepared to sustain. No good substitute for the leech has yet been, or, in our opinion, is ever likely to be, invented. Hence, every mean whereby the propagation of this valuable animal may be promoted among us, and the mortality which, at present, commits such wide depredations upon the species, diminished, deserves to be most attentively explored and as sedulously cultivated. These objects, it is evident, are only to be attained by close observation of its natural habits and propensities, and by correct acquaintance with its structure and physiology. Hence, our account of the *diseases and management of the leech* will be advantageously preceded by a review of the *natural history* and *anatomical structure* of the animal; and to the whole, a concise notice of its *medical history* may, with propriety, be prefixed. This, in fact, constitutes the judicious arrangement of Dr. Johnson.

---

* Imported leeches are principally supplied from Bourdeaux and Lisbon. Dr. Johnson asserts that, on a moderate calculation, we employ at least one hundred foreign leeches for every English one.

Section I. *The Medical History of the Leech.* Casual observation of the effects of the leech, in its attacks upon other animals, probably suggested the first idea of its introduction into medical practice; and experience of its beneficial operation on the human frame, when suffering from accident or disease, would alone be wanting to sanction and extend its employment. During the infancy of medicine, in the schools of Egypt and of Greece, the uses of the leech appear to have been unknown. Themison, disciple of Asclepiades, and founder of the methodic sect, who practised at Rome about the close of the reign of Augustus, or commencement of that of Tiberius, first notices them in his writings. He applied a cupping-glass over the orifice, formed by the leech, in order to augment the flow of blood. Of his success in the practice, nothing can be learned. Among the more celebrated authors, who subsequently wrote in commendation of the virtues of the leech, may be principally enumerated, Pliny the Naturalist, Aretæus, Ætius, Ægineta, and Zacutus Lusitanus. By these it was, for the most part, employed, or recommended, against topical pains and inflammation. In several forms of chronic and febrile disease, abstraction of blood, by its means, from the hæmorrhoidal veins, was deemed an almost unfailing remedy. If, in the present day, the intimate connection which subsists between those veins and the system of vessels constituting the portal vein and inferior cava, were better recollected, and the indications which it might suggest in diseases of the abdominal organs, more frequently acted upon, the reputation of the science and interests of the sick would, we are convinced, be, on many occasions, very materially promoted. Much too as the practice of the French physicians in this respect has been ridiculed and decried by some of our countrymen, it is equally certain, that applications of the leech to the external sexual organs of the human female, constitutes one of the most direct and unerring means whereby the sufferings to which she is exposed, from frequent disturbance of the functions of the uterine system, may be relieved. Zacutus records a case of phrenitis, consequent on retention of the menstrual flux; wherein, after the failure of the wonted remedies, the application of four leeches, properly secured by thread, to the vagina in the immediate vicinity of the orifice of the womb was followed by cessation of the phrenitic symptoms and speedy convalescence. Nor, in the inflammations, chronic or acute, of the serous membranes, lining the great cavities, is the decided superiority of sanguineous depletion by leeches, from the neighbourhood of the inflamed membrane, to the practice of venæsection, generally understood or sufficiently appreciated. Little difficulty would be encountered in tracing the medicinal employment of the leech down to the present day, but enough has been advanced to demonstrate its antiquity; and the evidence of succeeding ages has served only to exalt the character of the remedy, and fix it upon a surer basis.

Let us turn rather to contemplate the formidable accidents which may result from its employment, and consider the most prompt and effectual means of obviating the dangerous consequences sometimes induced by fortuitous introduction of the leech into the fauces, or intestinal canal, of the human subject. Such accidents have not unfrequently been witnessed; and in the writings of the ancients may be found a description of the symptoms denoting the presence of the leech in the throat or stomach, and an enumeration of the remedies best calculated to dislodge the terrible intruder. Of the latter, the principal are vinegar, garlic, salt water, assafœtida, mustard, and other acrid substances, copiously administered; sternutatories of elaterium and hellebore; or, when pain in the throat indicated that the animal was there, retention of cold water in the cavity of the mouth, whereby it might be decoyed into a situation less inaccessible and remote. But the celebrated French surgeon, Larrey, of all the authors with which we are acquainted, has given the best description of the phænomena resulting from the attachment of a peculiar kind of Egyptian leech to the faucial membrane, and of the method of treatment, by which the dangers of the accident may be averted. The introduction, therefore, of a brief abstract from the Baron's interesting memoirs,* will not be considered, by the reader, irrelavent to our subject, or destitute of instruction.

During the operations of the French army in Egypt, pools of soft and muddy water were frequently met with, particularly in the deserts bordering upon Syria. These contained, among other animals, a species of leech, which, though but a few millimetres in length, and fine as a

---

* *Memoires de Chirurgie Militaire, &c.* Tome i. page 359.—See also the recently published translation, by Mr. Waller.

horse-hair, acquired, when gorged with blood, the volume of an ordinary leech. It was black, and somewhat resembled the species which has been observed in the island of Ceylon. The soldiers, urged by thirst, and unsuspicious of danger, threw themselves down upon the margins of these lakes, and drank with avidity; but many soon experienced the baneful effects of the leeches which they had swallowed. Painful sense of stinging in the posterior fauces, frequent cough and expectoration of glairy mucus streaked with blood, and disposition to vomit, were the primary consequences of the bite. To these symptoms succeeded swelling of the throat; frequent hæmorrhages; difficulty of swallowing and respiration; pains in the breast; increased cough from the irritation excited by the tail of the animal on the borders of the glottis, and perhaps also by that of the effused blood upon its orifice; perceptible emaciation; loss of sleep and appetite; restlessness and agitation, sometimes, unless timely assistance were afforded, terminating in death. On examination of the throat of the first individual attacked by these symptoms, a leech, of the size of the little finger, was discovered in the isthmus of the fauces, and with difficulty extracted by the polypus forceps. Slight hæmorrhage succeeded the operation; and the patient soon recovered. In other instances, gargles of vinegar and salt water, injections of salt water, fumigations of tobacco and squill, were successfully employed to dislodge the leech from the posterior fauces. Sometimes the animal descended into the œsophagus or stomach, from whence, after remaining some time, it was, at last, detached by the use of diluted vinegar, or by the contraction of the viscus itself. M. Latour-Maubourg, chef de brigade, in drinking the water of a small lake on the deserts of St. Makaire, about one day's journey from the pyramids, swallowed two of these leeches; by which he was dreadfully harassed during the rest of his march, and reduced to the last extremity of weakness and exhaustion. The cough and bloody expectoration continued after his arrival at Cairo; and medicine, their origin not being suspected, served but to aggravate his symptoms. At length, the leeches were discovered; one of them extracted by forceps from the pharynx, and the other dislodged from the nasal cavities by salt-water injections. A leech, situated like this last, in another subject, was only distinguished from nasal polypus by its sudden retraction on the contact of the instrument employed by M. Larrey to detach it. Persons, who, in traversing these deserts, are under the necessity of drinking water infested by such animals, should previously pass it through strong linen; and, if possible, add to it a few drops of some kind of acid. Should a leech penetrate into the human stomach, it is highly probable that it could not long exist there; although the facts, observed by Dr. Johnson, warrant an inference, that the animal, until its vital principle became extinct, would resist the solvent action of the gastric fluid. Under these circumstances, copious ingestion of salt water, would, doubtless, accelerate its destruction.

The ancients, whenever the hæmorrhage, consequent on application of leeches, was unnecessarily protracted, relied for its suppression, on plugging the orifice with powdered aloes, bole armeniac, or sponge dipped in liquid pitch; but continued pressure with the finger, whenever the orifice is situated over a bone, will be found a much more direct and efficient mode of accomplishing this object; or, under other circumstances, especially if the bleeding prove very obstinate and menace dangerous consequences, the formation of an eschar by the employment of caustic may be had recourse to with almost invariable success. The idea that forcible extraction of the leech from its hold could not be safely attempted, is completely disproved by the unceremonious practice of the French army surgeon; and hence, the story of the death of the Roman Consul, Messalinus, in consequence of the teeth of a leech being left in the wound which it had inflicted, is probably incorrect.

Section II. *The Natural History of the Leech.* Under the genus *Hirudo*, belonging to the order of *Vermes externi* in the Linnæan arrangement, many species are comprehended; but much confusion has arisen from the introduction into it of several individuals of other families, differing essentially from the generic character, as delineated by the Swedish Naturalist.*

---

* The *Hirudo viridis*, of Dr. Shaw, the *H. alba* and *H. nigra*, of Mr. Kirby are now included in the genus *Planaria*. Dr. Johnson also constistutes a new genus, which, from the retractile tubular tongue of the animals composing it, is entitled *Glossiphonia* (γλωσσα, a *tongue*, and σιφων, a *tube*). The following is the generic character which he delivers: Corpus subovatum depressum, caput accuminatum, lingua tubulata, os caudamque alternè affigendo progrediens. Under this genus, he arranges the *Hirudo complanata* and *stagnalis*, by the respective titles of *G. Tuberculata* and *G. Perata*. He thinks also, that the *Hirudo Crenata*, of Mr.

*Hirudo. Character Generis.* Corpus oblongum subrotundum, anterius et posterius truncatum, muticum, cartilagineum, os caudamque dilatando progrediens. The genus may be divided into those which occupy rivers or stagnant waters, and those which inhabit the ocean.—To the three first species of the former division, *Hirudo Medicinalis, Sanguisuga,* and *Troctina,* our observations will be principally restricted.*

*H. Medicinalis.* Hirudo depressa nigricans, supra lineis flavis sex, intermediis nigro arcuatis, subtus cinerea nigro maculata. Oculi decem, more delineato. Long. Pollices tres. In stagnis—palludibus. Caput, quiescens, subrotundum, progrediens accuminatum. Os, quoad figuram mutabile, rimam triangularem plerumpque exhibens. *Cauda,* circularis, complanata, fibris carnosis e puncto centrali divaricatis.

*H. Sanguisuga.* Hirudo elongata fusco-viridis, subtus cinereo virens, maculis nigris. Oculi decem. Long. Pollices tres. In stagnis—locisque pallustribus.

*H. Troctina.* Hirudo elongata fusca, supra annulis aureis, maculas atras cingentibus, margine sub-flavo laterali, subtus flavo-viridis punctis atris. Oculi decem. Long. Pollices tres. In rivis — Piscibus crebrò adhærens.†

*Appellations of the Leech in various countries.* By the Greeks it was named βδελλα; by the ancient Romans, *Hirudo* (from *haurio,* to draw out); by the moderns, *Sanguisuga.* The French call it *Sangsue;* the Italians, *Sanguisuca;* the

---

Kirby, and the *H. circulans,* inhabiting the Thames, will be ultimately found to belong to this genus. Indeed, it appears probable, that the *H. Crenata,* though described by Mr. Kirby as possessing only *two* eyes, is identically the same as the ordinary *H. Complanata:* for, singularly enough, the figure which accompanies Mr. Kirby's description, is delineated with *six* eyes, and has, moreover, a *retractile tongue.* Such is the argument of Dr. Johnson. We have not paid to this subject attention sufficiently minute to render decisive our opinion upon the justice of his ideas, and the correctness of his classification.

* The remaining species of the *fresh-water* division here enumerated, are the *H. Nigra, H. Vulgaris, H. Tessulata, H. Lineata, H. Heteroclyta, H. Geometra,* and *H. Marginata.*—*H. Indica, H. Grossa, H. Hippoglossi, H. Branchiata, H. Muricata, and H. Verrucosa,* are the species found in the *ocean.*

† "I am not aware," says Dr. Johnson, "of this species having been hitherto described. I have denominated it *Hirudo Troctina,* from its resemblance, in regard to the coloured rings or spots, to the trout, and also from its being known and sold in the shops under the name of the Trout-leech. Owing to the great scarcity of the medicinal or striped leech, it has been latterly employed in medicine."

Germans, *Blutegel;* those of the Low Countries, *Lyche lake;* the Danes, *Blodigle;* and Poles, *Pijavvka.*

The medicinal Leech is common throughout Europe, more especially in the southern parts. It is often seen, in the south of India and America, to measure six or seven inches, twice the common length of the European Leech. The predominant colour of the animal varies according to the colour of the soil which forms the boundary of its habitation; but the spots or lines with which it is marked, are nevertheless permanent; and hence invariably denote the specific character. Of the motions and mode of progression of leeches, we need not pause to speak. During winter, they inhabit the deeper waters; during summer, the shallows. If the cold of the former season be very rigorous, or the drought of the latter excessive, they retire deep within the ground, leaving a small aperture to their subterraneous abode. They commonly re-appear about the close of March. In cold and cloudy weather, they do not quit the mud. Before a thunder-storm, they rise to the surface, and may be collected with facility. Though considerably affected by atmospherical vicissitudes, they certainly do not predict, with any thing like precision, the changes of the weather. Vigilant observation, made for some weeks on a vessel containing a number of leeches, will suffice to convince any one of the correctness of this assertion.

The fluids of fish and frogs constitute the food of the *H. Medicinalis* and *Troctina;* but neither earth-worms, nor the larvæ of aquatic insects, are attacked by them. The *H. Sanguisuga,* on the other hand, possesses a voracious appetite, and the faculty of ingesting solid aliment. When urged by hunger, it will seize on the medicinal leech, and even devour individuals of its own species. Dr. Johnson saw one of these gluttonous animals swallow whole, within a very short time, two of the *H. Vulgares,* or rivulet leeches; and, three days afterwards, one of them was rejected "in a *living* state," apparently having suffered but little injury from this aukward discipline; and few hours only elapsed ere it was again swallowed. Another of the *H. Vulgares* was observed to deposit several ova, at distinct intervals, after liberation from two hours' confinement in the intestinal canal of the *H. Sanguisuga.* Dr. Johnson also found, from constantly supplying two of these animals with the *H. Vulgares,* both living and dead, that, within thirty days, one of the former devoured fifteen, and the other twenty of the latter. During the whole period, the water, though occasionally renewed, was turbid, from the copious evacuation of thread-like fæcal matter. The digestive powers of the *H. Sanguisuga* appear to possess considerable vigour and activity; for, upon opening two of them, which had each, five days previously, swallowed a rivulet-leech, not a vestige of it could be discovered in the alimentary canal. This peculiarity constitutes a striking exception to the general law of tardy digestion in cold-blooded animals; and is elucidated by a view of the peculiarities of anatomical structure which distinguish the horse-leech: for the intestines of this species is more than double the width of the same organ in the Medicinal and Trout-Leech; nor is the stomach so thickly set with membranous folds or partitions. Analogy renders it probable, that the digestive organs of this voracious animal act with increased energy during the summer; and it evidently possesses the power, enjoyed by carnivorous birds, of rejecting at pleasure recently swallowed food.

The result of Dr. Johnson's numerous observations and experiments upon the two respective species of this very singular genus is, that the *medicinal Leech* takes no solid aliment; that, in its native abode, it subsists on the fluids of fish, frogs, &c.; that it possesses not the voracity which distinguishes the Horse-Leech; and that it betrays no propensity, like the latter, to destroy its own species, or any other belonging to the same genus. With respect to the *Horse-Leech*, that it devours alike the *H. Medicinalis*, *H. Vulgaris*, and even the weaker of its own species, in the absence of other alimentary substance; and will swallow almost any thing within its reach. Hence the designation of *H. Vorax* is more characteristic of, and correctly applicable to, the species, than that of *H. Sanguisuga*.

Leeches have been said to possess the extraordinary property of regeneration, when mutilated, or even divided into several portions. The experiments of Dr. Johnson render it probable that this statement is incorrect.

With regard to its mode of propagation, the Leech is decidedly hermaphrodite, and has repeatedly been observed in the act of double copulation; but, although the generative function is sometimes exercised during confinement, it seldom produces young in that state. Upon the question, whether the animal be oviparous or viviparous, much difference of opinion exists. Some naturalists, of high celebrity, state, that young are produced, resembling, in every respect but size, the parent leech; and this inference derives additional support from the structure of the uterus, and largeness of the genital organs. By others it is, with equal confidence, asserted, that the animal deposits ova, which are subsequently hatched. Certain it appears, that the *Coccus Aquaticus* is the ovum of the *Hirudo Vulgaris*;* and an emphatic *vidi et obstupui* burst from the lips of the wonder-stricken Linné, as, on conviction of the fact, his ingenuous mind renounced its previous error. Dr. Johnson inclines also to the opinion, that the medicinal Leech is oviparous. On renewing the water, in which some of this species were preserved, he remarked an ovum attached to the side of the vessel. It was semi-oval, furnished with a brown shell or crust, and transparent; larger than the ovum of the *H. Vulgaris*, but, like this, when deposited in a state of confinement, unproductive, although kept for many months. Yet, if the mass of conclusive evidence, which has been adduced on the opposite side of the question, be duly and dispassionately considered, we absolutely descry no other mode of solving the difficulty, and reconciling the contradictory statements, than by admission of Dr. Johnson's probable conjecture, that, under certain circumstances, the medicinal Leech is, like the Aphides, and several other insects, both oviparous and viviparous.

The Leech is exceedingly tenacious of life. Not only will it exist, when deprived of its head or otherwise mutilated, for several months, but sustain little apparent injury or inconvenience from confinement, of many days, beneath the exhausted receiver of an air-pump. A leech thus circumstanced, has been known to support the state of insulation more than three weeks. It is, moreover, demonstrated by experiment, that the animal will live in a glass vessel, containing three cubic inches of hydrogen gas, 2 days 12 hours; a like quantity of carbonic acid gas, 5 hours; of nitrogen gas, 8 days; of atmospheric air, 10 days; oxygen, 12 days; water, impregnated with carbonic acid gas, 4 hours; olive oil, 1 day 16 hours; spring water, the vessel being stopped, 7 days. The organs of the young leech are exceedingly tardy in their development. About five years are supposed to elapse ere they attain a state of maturity. It is probable that the animal will live in its native waters, with an abundant supply of food, at least twenty years.

* Each ovum yields, on being hatched, from one to ten young.

SECTION III. *The anatomical Structure of the Leech.** We have now to contemplate the Leech, first, in its *external structure*; secondly, in the constitution of its *sentient and respiratory organs*; and, thirdly, its *internal structure*. For the sake of brevity, we shall endeavour to introduce into this part of our article the concise and rigorous character of botanical description.

*External Structure.* *Body,* when quiescent, arched, and in length more than an inch; capable, when moving, of great extension; furnished with rings or annular muscles, usually about one hundred, increasing in size, not in number, with age, and each presenting, on its most prominent part, a row of minute tubercles. Mouth, of a circular or horse-shoe figure, but capable of assuming any other; external surface dark grey; internal, light grey: upper lip slightly bent downward with a central cleft; lower, bent inward, and, like its fellow, semi-circular. Sucker, at the oral extremity,† formed by fasciculi of muscular fibres, some disposed in a radiated, others in a circular direction. Caudal sucker, similarly constituted. First generative orifice, situated in the abdomen, half an inch from the lower lip, round, small, affording passage to the male organ of generation: ‡ second orifice, or vagina, leading to the uterus, distant about five rings from the former, towards the caudal extremity, and with difficulty perceptible: third orifice, or anus,§ placed on the back, very near the rim of the caudal sucker, and capable of admitting a pin, although smaller in the *H. Medicinalis* and *Troctina,* than in the *H. Sanguisuga.*

ORGANS OF THE SENSES. *Organ of vision.* Eyes ten in number; ‖ arranged in a crescent shape, at the pointed extremity on the back of the head, the two at each ex-

---

* Two engravings, correct as neatly executed, illustrate this description.

† We have taken the liberty of substituting the more precise terms of *oral* and *caudal* sucker, for superior and inferior. They constitute the two extremities of the animal; and by their means it is capable of very firmly attaching itself to any surface.

‡ This has been mistaken for a respiratory orifice; but the penis may frequently be seen protruding from it, particularly in the dead leech.

§ By many celebrated naturalists, the leech has been described as destitute of an anus; but the inaccuracy of this statement is proved by the fact, that a fluid may be so injected through the mouth of the animal as to issue in a stream from the dorsal orifice, and vice versâ. Fæcal matter may also, by compression of the body of the leech, be forced out of this opening.

‖ This description applies only to the *H. Medicinalis, Sanguisuga, Troctina,* and *Nigra.* They "who wish to have preparations of these tremity of the arch, more distant from each other than the rest; deep black; when moistened, possessing a fine lustre, and seen beneath the microscope as tubercles jutting from the skin. *Organ of touch,* residing in the lips, and perhaps in the disk which terminates the caudal extremity. *Of taste,* composed of an assemblage of nervous fibrillæ situated in the upper part of the œsophagus. *Of hearing,* none yet discovered. *Of smell,* probably will be found to exist in the puncta respiratoria, by which the leech is very confidently believed to breathe.

*Respiratory organs.* The opinion of naturalists on this subject is much divided. Some contend that the Leech respires by the mouth; others, among whom is Dr. Johnson, that the process is executed by means of spiracula or breathing holes.*

*Internal structure.* An epidermis, cutis, muscular and membranous coats, with the interposed cellular substance, constitute the coverings of the Leech. *Epidermis,* or outer membrane, thin, fibrous, reticulated; cast off every fourth or fifth day, and seen, like a small ring, floating in the water. *Cutis,* externally dense and firm; internal surface flocculent; texture spongy; firmly attached to the subjacent muscular coat, especially in the inter-annular spaces, where much of the flocculence disappears. *Muscular coat,* ash grey, stong, elastic, consisting of two strata of fibres, circularly and longitudinally disposed, interlaced and inseparable without injury; the longitudinal very conspicuous. *Membranous coat,* delicate, lining the whole internal cavity; exhibiting, beneath the microscope, the appearance of fine lace.

On dividing the belly of the Leech, in a direct line from mouth to anus, but not so deeply as to implicate the alimentary canal, the following organs will be exposed to view: *Three piercers,* incorrectly named teeth, cartilaginous, rounded; furnished with sharp cutting edges; resting on small eminences, and so relatively placed as to meet, under equal angles, in a centre; confined by a strong circular ligament, which surrounds the œsophagus; possessing, when in action, an oscillatory movement, and

---

organs, may readily form them, by cutting off the head of the leech, and subjecting it, between two strong plates of glass, to continued pressure." Dr. JOHNSON.

* Thomas (*Memoire sur les Sangsues*) has mistaken certain membranous sacs, which contain an unctuous fluid destined to lubricate the surface, for the respiratory organs. Of these we shall hereafter speak.

inflicting a triangular wound. *A nervous mass or brain,* consisting of a ganglion, which surrounds the œsophagus, and sends off filaments to various parts, particularly two lateral nerves proceeding to the tail, and here and there expanding in their course: and a central nerve, occupying the *interior* of the abdominal blood-vessel, and forming diamond-shaped ganglia, which correspond to a similar formation in the abdominal vessel.* *The blood-vessels:* An abdominal blood-vessel, pursuing a direct line from mouth to tail, enclosing the central nerve, and presenting in its course several diamond-like expansions; the first of these observed just below the lip; the second, third, and fourth, respectively, at the origin, middle, and termination of the œsophagus; the fifth, on the bag of the male generative organ; the others equi-distant, and ending close to the disk of the caudal extremity: two lateral vessels, nearly straight when the leech is in motion; when quiescent, assuming an appearance of a circle of festoons; and, lastly, a dorsal vessel, seated on the back, and passing directly from head to tail. All these contain red blood, and communicate by numerous transverse branches, of irregular distribution.† *The male generative organ,* a strong elastic tube, extremely irritable, enclosed in a circular bag, flattened above and below, about one inch in length, often seen hanging from the first generative orifice. *The testes,* oblong bodies, containing a thick greyish white fluid, placed at the commencement of the first pair of cells or stomachs, on each side of the male organ, and transmitting by tubes their contained fluid to the generative bag.‡ Several oval bodies *(the abdominal vesicles),* of the size and colour of mustard-seed, placed in pairs on the surface of the cells or stomachs (the first pair near the testes, the others equi-distant and ending about the middle of the last stomach), connected to the testes by a tortuous tube, and containing a similar fluid. *The female generative organ,* an oblong sac, somewhat resembling in figure a bagpipe, composed of a strong

* By Hunter, Poupart, and Cuvier, the abdominal blood-vessel, presently to be described, has been erroneously represented as the main organ of the nervous system.

† A distinct systole and diastole is perceptible in these vessels on opening the *H. Medicinalis alive,* or holding up to the light the *H. Vulgaris.* In the former, Dr. Johnson has counted ten; in the latter, eight of these pulsations in the minute.

‡ Some writers have regarded these as a nervous mass or brain, and the bag of the male generative organ as an uterus.

membrane covered with muscular fibres; connected with the vagina, the orifice of which has been described; and furnished posteriorly with an oviduct leading to the ovaries: it is endowed with a strong peristaltic motion. *The lateral vesicles,* membranous sacs, about thirty in number, occupying the site of every fifth ring, on the sides of the body, and containing the unctuous fluid for lubrication of the surface. *The œsophagus,* about one fourth of an inch long; at first narrow, gradually widening, and again contracting as it reaches the first pair of gastric cells; furnished internally with several longitudinal muscular folds, whereby the motions of the piercers are regulated, externally, with several strong muscular bands: *The cells,* twenty-two in number, or rather a *single stomach* divided into so many partitions; formed by reflection of the internal membrane, and constituting four-fifths of the bulk of the animal; semi-oval in figure, except the two last, which are oblong and terminate in blind sacs close to the anal extremity*: *The alimentary canal,* a continuation of the œsophagus, about the size of a crow-quill; furnished with openings on each side, which lead, and correspond to the several cells, and throughout with membranous folds, which perform the office of valves in preventing a return of the alimentary matter: And, lastly, *the intestine,* about an inch in length; situated between the two last long cells, and confined by two ligamentous bands obliquely directed at its upper part; furnished at the gastric orifice with a valve which precludes regurgitation of the fæcal matter, commonly filling it, into the stomach; at the anal extremity, with a sphincter which opposes the escape of blood per anum in an overgorged leech. The diameter of this canal is small in the *H. medicinalis.*

SECTION IV. *The disesases, preservation, and management of the Leech.* The leech is said, like other animals, to be infested with lice: and from the frequent occurrence of the following forms of disease, great mortality prevails among the species: 1. An ulcer, variously seated, commonly on the side, presenting at first, only a small ulcerous speck, afterwards spreading in a very rapid and ma-

* The remarkable structure of the stomach may be advantageously exhibited by immersing a leech, when fully gorged with blood, for a week or ten days, in saturated solution of muriate of quick-silver, and afterwards opening it and picking out with a needle the contents of each cell: or, by filling the cells of the recent dead animal with crude mercury, the precise figure of each will be more clearly demonstrated.

lignant manner; sometimes superficial, sometimes burrowing internally; often tinged with blood; very destructive: the part, constituting its seat, usually contracted.* 2. Another affection, equally malignant, wherein one portion of the body is contracted in diameter and rigid, and another studded with tumors presenting, on incision, black putrid, coagulated blood: 3. A general flaccidity of the body, with exception of the lips, which are indurated, swollen, purple, often bloody. These diseases do not exclusively affect the leech in a state of confinement; prevail particularly during summer; are contagious, and invariably fatal. Hence, any leech, exhibiting signs of indisposition, should be immediately secluded from the rest. Should the water, containing the leeches, display a bloody tinge, an impending mortality is denoted; and it will be prudent to separate, and keep them in small numbers, even although apparently healthy.

Leeches, when in considerable numbers, should be *kept* in a large stone vessel furnished with a false bottom, so perforated as to allow the passage of the animals, and with a stop-cock whereby the water may be drawn off. The false bottom should be so elevated, as to allow the introduction of a turf, or quantity of marsh horse-tail *(equisetum pallustre)* between it and the real one.† The water should be preserved of a regular temperature; changed about once a week; and the situation, chosen for the jar, be free from all unpleasant smell. Previously to introduction, each leech should be narrowly inspected; and all those, the body of which feels flabby, or exhibits protuberances or white superficial ulcerated specks, should be kept separate, and have the turf and water of the containing jar frequently renewed.

The part on which leeches are to be applied, should be washed perfectly clean, first with soap and water, then with water alone. Removed one hour previously from the water, they may be affixed by inclosing them in a wine glass, leech glass, or perhaps most conveniently by the hand. Should any difficulty be experienced, a slight puncture may be made in the skin with a lancet, or the surface besmeared with a little blood taken from the ori-

* This ulceration is particularly common in the *H. vulgaris.*

† By these means, the leech is enabled to rid itself of the epidermis, which, unduly retained, might prove a source of disease. During the early part of spring, leech-catchers employ the *equisetum pallustre*, in a dry state, to allure the leeches to the surface of the water.

fice of a leech already fixed, or from some other source. The animal, if indolent in its office, may be effectually roused by sprinkling with cold water: and its separation, if in the event of proving very tardy, be accomplished by insinuating the finger nail between its mouth and the surface, to which it is attached. Whenever a sufficient number of leeches cannot be procured, the ancient practice of cutting off, or making a deep incision close to the tail of such as are employed, may be conveniently adopted. Thus, the blood will continue to ooze from the wound as fast as it is swallowed by the animal: and any quantity required may be drawn away. But the operation will only succeed when performed while the leech is in the act of suction: for, in the absence of the pressure from the descending column of blood, the artificial orifice would be closed by the strong contraction of the circular muscles. The animal, though at first apparently suffering but little, eventually becomes very languid from this operation; which, therefore, should never be had recourse to, except on the spur of necessity. When fully gorged, the leech contains from half an ounce to an ounce of blood; and, singularly enough, this will remain uncoagulated in the stomach for two or three months, without yielding any offensive smell. It merely acquires a deeper colour, and thicker consistence. Its coagulation, under certain unknown circumstances, is productive of disease. A sprinkling of muriate of soda is commonly employed to make the leech disgorge; but, by this mean, the body and lips are blistered, and the animal rendered incapable of reapplication for some days. Hence we are recommended to expel the contained blood by gentle pressure between the thumb and finger. To this mode of evacuation the risque of injuring the internal structure of the animal constitutes a serious objection. The affusion of a little vinegar on the head, is far preferable. Thus treated the leech may be made to fill itself three or four times in succession. It should be allowed to retain for support about one third of the blood extracted. Lastly, in order to prevent imposition and mistake, we may observe, that no leech should be employed which is not marked with yellow rings or spots, or with variegated lines running the whole length of the back.

We shall, perhaps not inappropriately, conclude this article by earnestly recommending it to all young men, destined for the profession of medicine, or actually engaged in it, the study of natural history. The attention of the former cannot be too early directed to it. Far more profitably will the ardour of youth be thus occupied and expended, than in enervating the mind with the luscious poison of poetic fiction and romance, or cultivating, at a sad expence of time and intellect, the external graces: nor will the practitioner find his season of leisure less usefully employed in these pursuits, than in attendance at the feast, the card-table, or the midnight revel; or in the exercise of those paltry arts by which the ephemeral reputation of the fashionable physician is too commonly acquired. Studies like these, not only enlarge the sphere of intellect, give to the hour of retirement an unspeakable charm, and solace, and exalt and dignify, in public estimation, the medical character: they serve to generate or improve that taste for patient research, those habits of close and correct observation, without which, genius, erudition, labour, will avail but little, in gaining a profound and intimate knowledge of the phænomena of diseases, or in directing the practical physician, with honour to himself and safety to his patients, through the obscure and intricate paths with which the territory of medicine abounds. Shall he, who professes to understand the conditions of health and disease in the noblest and most mysteriously constructed fabric on the field of creation, restrict his enquiries to a narrow limit, and slight the instructive views which extended observation of the simpler forms of organized beings cannot fail to disclose to the philosophic eye? Shall he, who aspires to regulate the aberrations, and repair the injuries, of the human organs, content himself with a coarse and superficial knowledge of the plants, the animals, and varied productions of the inorganic world, which constitute the resources of his art; and the operation of which he is incessantly called upon to modify and direct?

PART III.

